# Stem cell-derived exosomes preserve diabetic islet function: a systematic review and meta-analysis of preclinical studies

**DOI:** 10.52601/bpr.2026.250071

**Published:** 2026-08-31

**Authors:** Yingling Jiang, Lihua Chen, Haopeng Lin, Tongran Zhang, Xin Liu, Zhihua Dou, Xu Wei, Lizhe Cai, Yan Hu, Huisheng Liu, Yanying Guo

**Affiliations:** 1 Graduate School, Xinjiang Medical University, Urumqi 830054, China; 2 Department of Endocrinology and Metabolism, People's Hospital of Xinjiang Uygur Autonomous Region, Xinjiang Clinical Research Center for Diabetes, Xinjiang Endocrinology Diabetes Institute, Xinjiang Key Laboratory of Cardiovascular Homeostasis and Regeneration Research, Urumqi 830001, China; 3 Guangzhou National Laboratory, Guangzhou 510005, China; 4 State Key Laboratory for Development and Utilization of Forest Food Resources, Zhejiang A & F University, Hangzhou 311300, China; 5 School of Biomedical Engineering, Guangzhou Medical University, Guangzhou 511436, China; 6 Shenzhen University Medical School, Shenzhen University, Shenzhen 518055, Guangdong, China; 7 Department of Endocrinology and Metabolism, The Affiliated Hospital of Southwest Medical University, Luzhou 646000, Sichuan, China

**Keywords:** Stem cell, Exosomes, Diabetes, Islet function, Meta-analysis

## Abstract

Diabetes remains an incurable disease, and emerging evidence suggests that exosomes may offer promising therapeutic potential for both diabetes and its complications. However, their specific effects on pancreatic islet function are still under investigation. This meta-analysis aimed to evaluate the efficacy of stem cell-derived exosomes (SC-Exs) in preserving islet function in diabetes. A comprehensive search of PubMed, Embase, Cochrane Library, and Web of Science databases was conducted from inception to May 1, 2025, to identify studies assessing the impact of SC-Exs on islet function in diabetic models. Data on insulin levels, islet quantity, and inflammatory markers were extracted and analyzed using Stata 14.0 and Review Manager (RevMan) 5 software. Ten eligible studies were included in the meta-analysis. Compared with controls, SC-Exs significantly increased insulin levels (SMD = 10.09, 95% CI: 7.97–12.21, *P* < 0.00001) and islet quantity (SMD = 3.33, 95% CI: 1.47–5.20, *P* = 0.0005). Moreover, SC-Exs consistently promoted islet proliferation, inhibited cell death, and suppressed inflammatory responses in diabetic models. These findings suggest that SC-Ex treatment effectively preserves pancreatic islet structure and function, highlighting its potential as a novel and promising therapeutic approach for diabetes management. Further high-quality studies are warranted to confirm its efficacy, safety, and clinical applicability.

## INTRODUCTION

Diabetes mellitus (DM) is a chronic metabolic disease characterized by hyperglycemia resulting from defective insulin secretion, impaired insulin action, or both (Anel *et al*. 2019). DM has become a major global public health challenge, imposing substantial long-term burdens on patients and healthcare systems. According to the International Diabetes Federation (IDF), an estimated 537 million adults were living with diabetes worldwide in 2021, and this number is projected to rise to 643 million by 2030 and 784 million by 2045. In addition, diabetes and its complications were associated with approximately 6.7 million deaths in 2021, accounting for about 12.2% of all-cause mortality, underscoring its enormous societal impact (Sun *et al*. 2022). Diabetes is a metabolic disease characterized by chronic hyperglycemia, for which no curative therapy is currently available. Current management largely focuses on glycemic control and complication risk reduction through lifestyle intervention and pharmacotherapy, but these approaches are generally not disease-modifying and long-term control remains challenging due to disease progression, heterogeneous responses, treatment burden and adverse effects, adherence barriers, and cost/access constraints. Although remission or insulin independence can be achieved in selected situations (*e*.*g*., metabolic surgery or pancreas/islet transplantation), these options have limited generalizability and practical constraints. Therefore, there is an ongoing need for novel therapies with more durable and potentially disease-modifying benefits, which motivates the present study. Currently, diabetes management relies on lifestyle interventions in combination with pharmacotherapy (Davies *et al*. 2022). Although available glucose-lowering medications can significantly improve glycemic control and reduce the risk of complications, they often require long-term administration and may be limited by adverse effects (*e*.*g*., hypoglycemia and gastrointestinal discomfort), suboptimal adherence, and variable treatment responses. More importantly, many current therapies primarily address hyperglycemia rather than fully preventing or reversing progressive β-cell dysfunction and loss, which contributes to ongoing disease progression and residual cardiometabolic risk (DiMeglio *et al*. 2018), highlighting significant challenges and the urgent necessity for developing novel therapeutic strategies.

Pancreatic islets, particularly insulin-secreting β-cells, are central to glucose homeostasis through tightly regulated insulin production, processing, storage, and secretion. Islet dysfunction — manifested by impaired insulin secretory capacity, loss of β-cell mass, and disrupted hormone regulation — represents a core pathological feature of diabetes and directly contributes to chronic hyperglycemia and glycemic variability. Therefore, preserving islet integrity and restoring β-cell function (including insulin granule integrity and secretory function) are increasingly recognized as critical therapeutic objectives (Li *et al*. 2018; Lin *et al*. 2024a).

Exosomes are small, membrane-bound vesicles secreted by various cells and are widely present in various body fluids. They are recognized for their role in intercellular communication. As a cell-free therapy, exosomes have emerged as a promising novel approach for the treatment of diabetes mellitus and its complications (Ashrafizadeh *et al*. 2022). However, the effects of exosomes on pancreatic islet function in diabetes remain controversial. Some studies indicate that mesenchymal stem cell (MSC)-derived exosomes can reduce cell death of pancreatic islet β-cells, thereby promoting the recovery of insulin secretion function (Chen *et al*. 2020). However, other studies have reported contrasting findings, where β-cell-derived exosomes significantly upregulate exosomal microRNA-associated signals, which in turn reduce the protein expression of insulin-related signaling molecules and lead to dysregulation of glucose-stimulated insulin secretion in β-cells (Yu *et al*. 2024).

These discrepant findings may, at least in part, be explained by differences in exosome origin and cargo composition. Exosomes from distinct sources carry variable repertoires of cytokines, trophic factors, signaling molecules, and nucleic acids (*e*.*g*., mRNA, microRNA, and long non-coding RNA), which can lead to divergent biological effects. (Noonin and Thongboonkerd 2021). Notably, accumulating evidence indicates that exosomes derived from stem cells tend to exhibit beneficial and protective effects in diabetic settings (Ren 2019). To substantiate the impact of SC-Exs on islet function and evaluate their potential therapeutic effects on diabetes, we conducted a meta-analysis to elucidate the relationship between SC-Exs and islet function.

## RESULTS

### Search results

**Figure 1:**
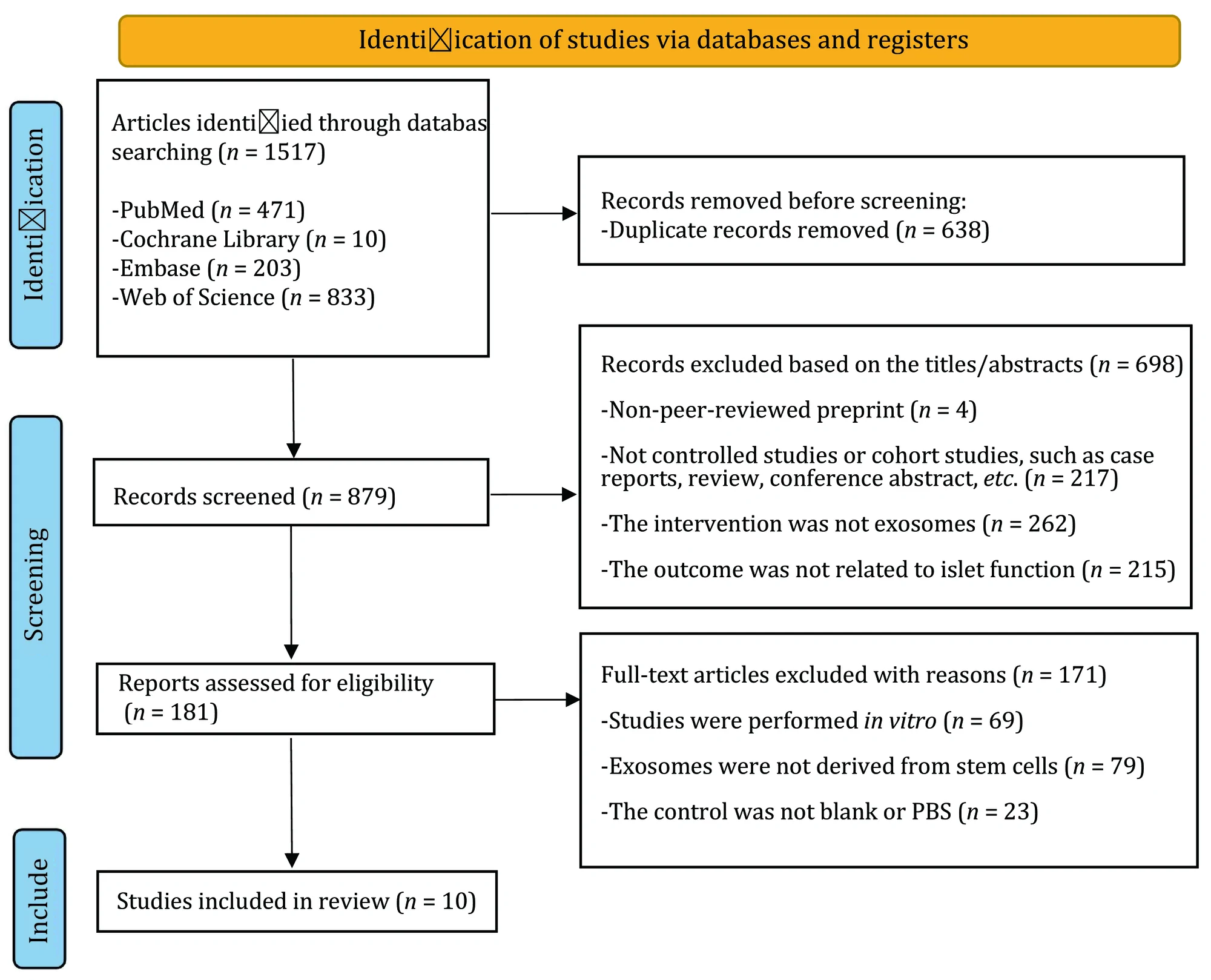
PRISMA flow diagram of literature search and selection of studies for meta-analysis

As shown in Fig. 1, a comprehensive literature search identified 1517 potentially relevant articles. Of these, 638 were removed as duplicates. After screening titles and abstracts, an additional 715 articles were excluded. Finally, a thorough review of the full texts of 50 research articles led to the inclusion of ten studies (Kashani *et al*. 2023; Kouhestani *et al*. 2023; Mahdipour *et al*. 2019; Nojehdehi *et al*. 2018; Ou *et al*. 2024; Sabry *et al*. 2020; Sharma *et al*. 2021; Sun *et al*. 2018; Wang *et al*. 2023; Xia *et al*. 2024) in this meta-analysis.

### Characteristics and quality assessment

**Figure 2:**
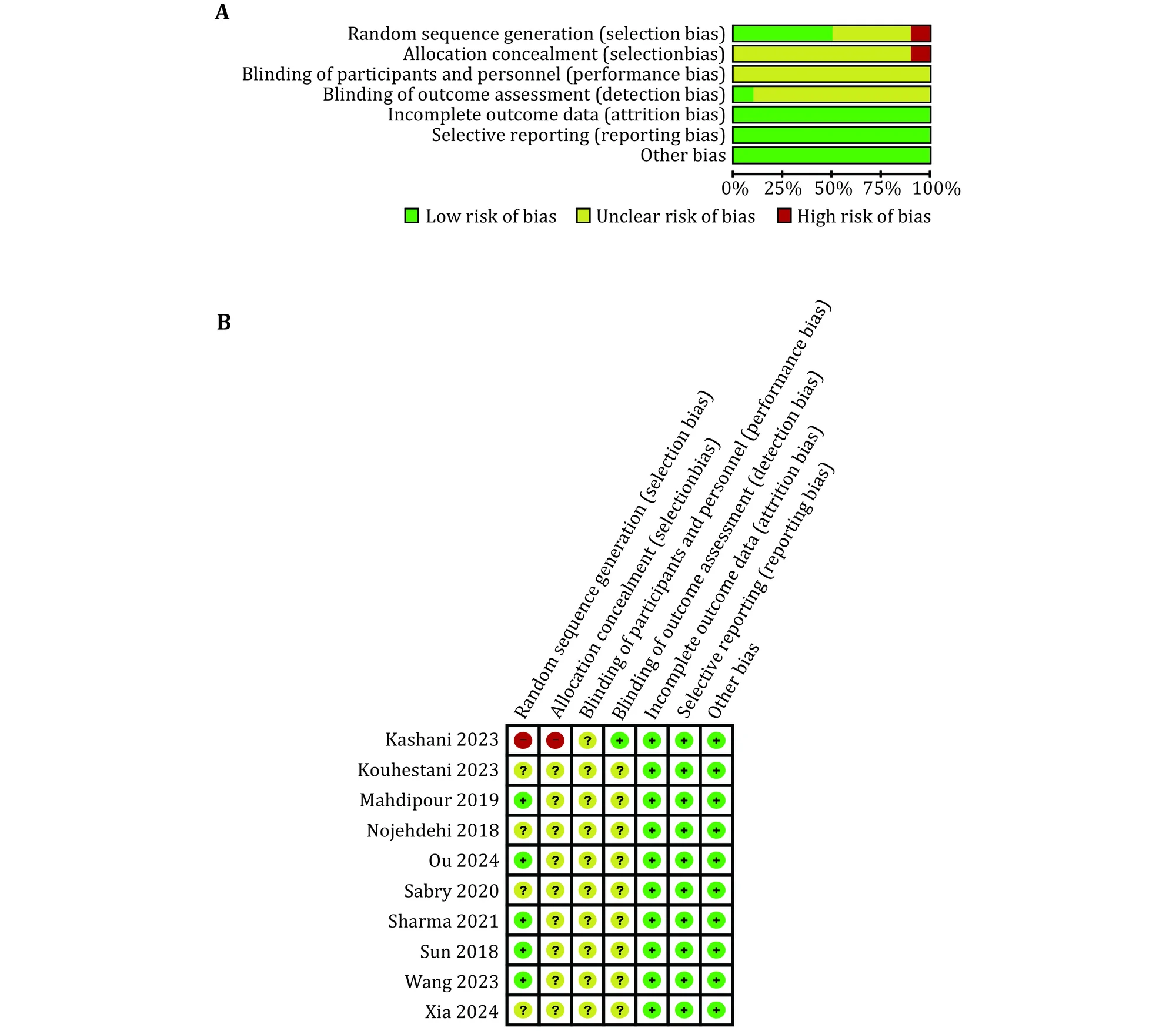
Summary of the bias risk of the studies. **A** Risk of bias summary across various domains for each study included in the analysis. The figure shows individual risk assessments for random sequence generation, allocation concealment, blinding of participants and personnel, blinding of outcome assessment, incomplete outcome data, and selective reporting. Green circles represent low risk of bias, yellow circles indicate unclear risk, and red circles indicate high risk of bias. **B** Overall risk of bias for each study, showing a distribution of bias across the included studies

Table 1 summarizes the studies included in the analysis. All studies, published between 2018 and 2024, were preclinical and conducted in animal models (mice, rats, or monkeys) with sample sizes ranging from two to eight per group. Exosomes were derived from five stem cell sources — BMSC, MMSC, ESC, UCMSC, and ADSC — using varying doses. Follow-up periods ranged from one day to two months. Five studies reported random allocation, four did not specify the method, and one explicitly stated that randomization was not performed. Study quality assessments are detailed in Fig. 2.

**Table 1:** Summary of the characteristics of the studies

Study	Year	Experimental animals	Source of exosomes	Administration route	Treatment cycle	Dosing frequency	Duration of follow-up
Kashani	2023	Rhesus monkeys (Male and Female)	BMSC (human)	Celiac artery injection	14 days	Every 2 weeks	55 days
Kouhestani	2023	Wistar Rats (Male)	ESC(human)	Micro-ultrasound-guided pancreatic injection	Not mention	Not mention	8 weeks
Mahdipour	2019	Wistar Rats (Male)	MMSC (human)	Tail vein injection	9 days	8 days apart	30 days
Nojehdehi	2018	C57Bl/6 mice (Male)	ADSC (human)	Intraperitoneal injection	2 months	Twice a week	8 weeks
Ou	2024	NOD/Ltf mice (Female)	UCMSC (human)	Tail vein injection	8 weeks	twice a week	8 weeks
Sabry	2020	Albino rats (Female)	BMSC(rat)	Intraperitoneal injection	1 months	Once a week	4 weeks
Sharma	2021	C57Bl/6 mice (Male)	UCMSC (human)	Tail vein injection	7 days	Every day	1 day
Sun	2018	Sprague-Dawley rats (Male)	UCMSC (human)	Tail vein injection	15 days	Every 3 days	2 weeks
Wang	2023	C57Bl/6 mice (Male)	UCMSC (human)	Tail vein injection	16 days	Every 2 days	16 days
Xia	2024	C57BL/6J mice (Male)	UCMSC (human)	Tail vein injection	6 weeks	Twice a week	8 weeks
BMSC, bone marrow mesenchymal stem cell; MMSC, menstrual blood-derived mesenchymal stem cell; ESC, endometrial stem cell; UCMSC, umbilical cord-derved mesenchymal stromal cell; and ADSC, adipose tissue-derived mesenchymal stem cell							

### Stem cell-derived exosomes can increase insulin levels in experimental animals

Eight trials (Kashani *et al*. 2023; Kouhestani *et al*. 2023; Mahdipour *et al*. 2019; Ou *et al*. 2024; Sabry *et al*. 2020; Sun *et al*. 2018; Wang *et al*. 2023; Xia *et al*. 2024) with a total of 82 animals reported insulin/C peptide data for their experimental and control groups. The *Q-*test and *I*^2^-test demonstrated significant heterogeneity across studies (*P* < 0.05, *I*^2^ = 86% > 50%). Results from the random-effects model were *SMD* = 9.3, 95% *CI*: 4.73–13.86, *P* < 0.0001 (supplementary Fig. S1A).

Considerable heterogeneity was observed among the included studies. The Galbraith plot identified one study (Kashani *et al*. 2023) that markedly deviated from the regression line (supplementary Fig. S1B), suggesting it as a potential outlier contributing to heterogeneity. Upon further examination, the major difference between this study and the others was the experimental animal species, as it used monkeys, while all other studies were conducted in rodents. After excluding this study, the direction of the pooled effect remained consistent, and heterogeneity was markedly reduced (*I*^2^ decreased from 86% to 0%). In addition, the leave-one-out sensitivity analysis (supplementary Fig. S1C) demonstrated that the overall effect estimate was robust, as the pooled results did not change substantially when any single study was omitted.

**Figure 3:**
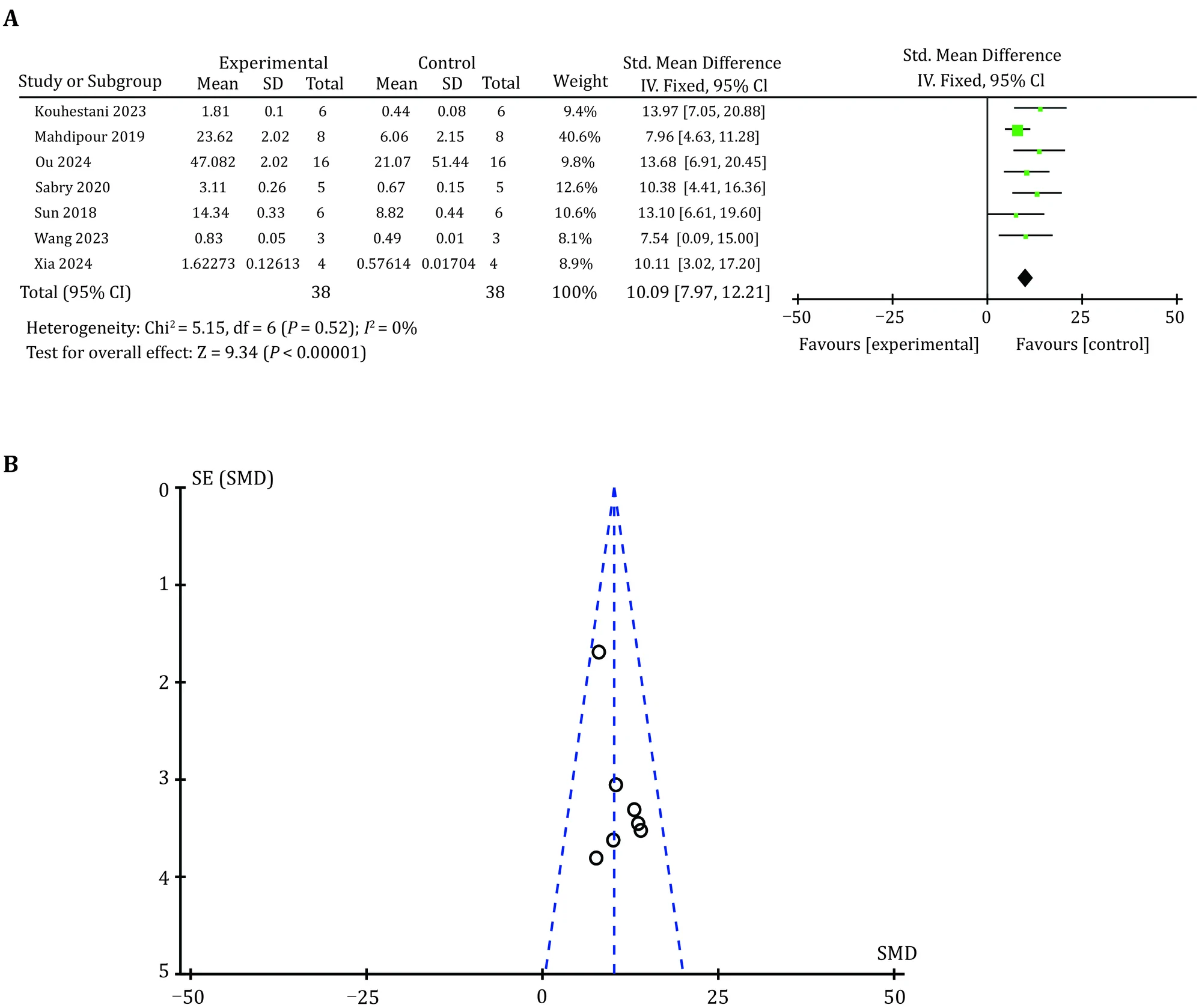
Meta-analysis of the effect of stem cell-derived exosomes (SC-Exs) on insulin levels after exclusion of the outlier study. **A** Forest plot showing the SMD and 95% CIs for insulin levels between SC-Ex–treated and control groups after removing the outlier study (Kashani *et al*. 2023). A fixed-effects model was used due to the absence of heterogeneity (*I*^2^ = 0%), and the overall results remained statistically significant. **B** Funnel plot displaying a symmetrical pattern, suggesting no apparent publication bias

The remaining seven studies were then included in the meta-analysis using a fixed-effects model. The pooled analysis showed a significant overall effect (*SMD* = 10.09, 95% *CI*: 7.97–12.21, *P* < 0.00001), as illustrated in the forest plot (Fig. 3A). These results imply that SC-Exs significantly improve insulin levels in diabetic animals. The funnel plot of this study is basically symmetrical (Fig. 3B), suggesting no obvious publication bias.

### Stem cell-derived exosomes increase the quantity of pancreatic islets in experimental animals

**Figure 4:**
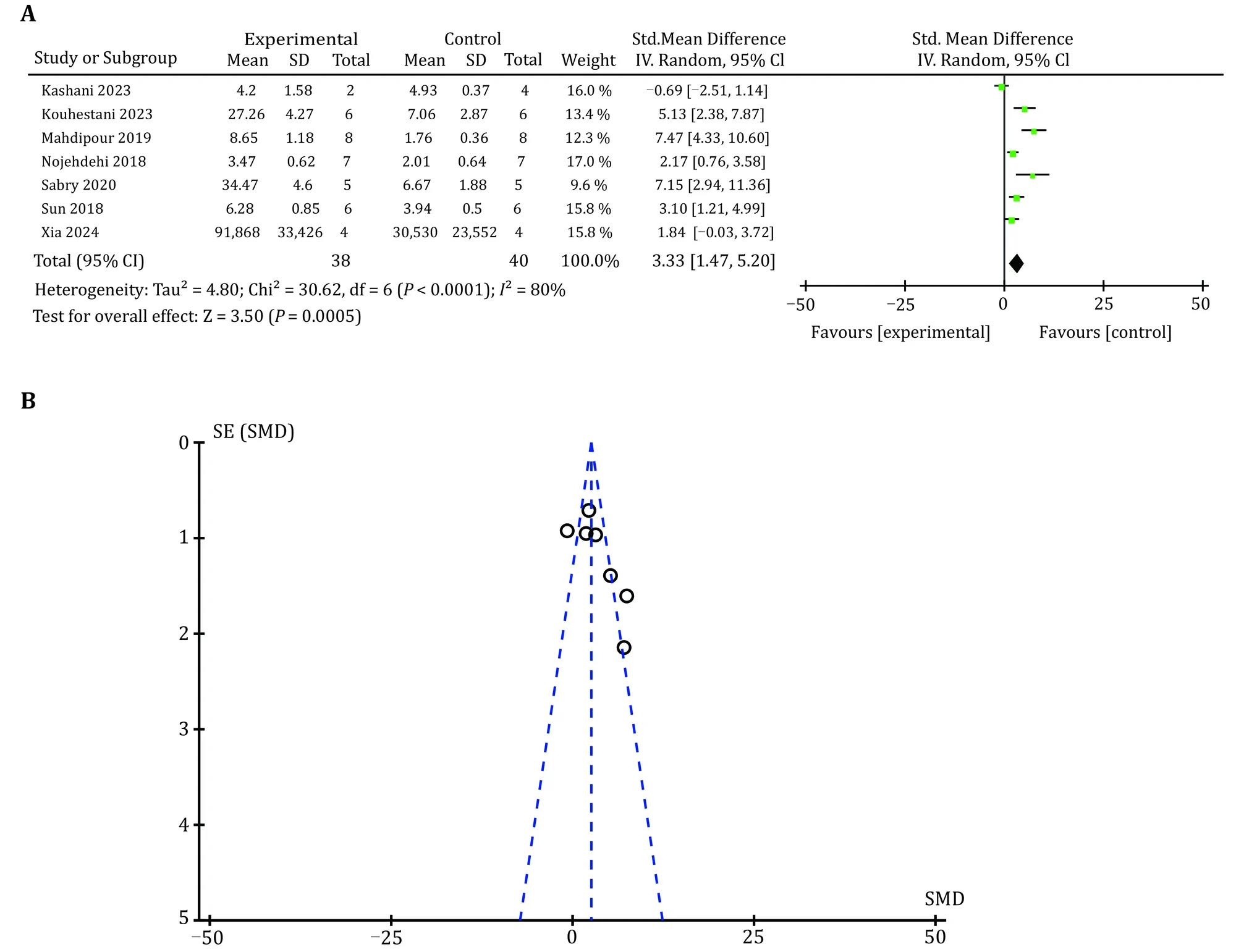
Meta-analysis of the effect of stem cell-derived exosomes (SC-Exs) on islet quantity in diabetic models. **A** Forest plot showing the SMD and 95% CIs for islet quantity between SC-Ex–treated and control groups, indicating a significant increase in islet quantity following SC-Ex treatment under a random-effects model. **B** Funnel plot assessing publication bias among the included studies, showing an approximately symmetrical distribution without substantial publication bias

Seven studies involving 78 animals have reported the effect of SC-Exs on the quantity of islets (Kashani *et al*. 2023; Kouhestani *et al*. 2023; Mahdipour *et al*. 2019; Nojehdehi *et al*. 2018; Sabry *et al*. 2020; Sun *et al*. 2018; Xia *et al*. 2024). In light of the considerable heterogeneity among the studies in question, and the source of heterogeneity could not be clearly identified, a random-effect model was used for meta-analysis. The results of this analysis indicated that the experimental group exhibited a greater number of islets than the control group (*SMD* = 3.33, 95% *CI*: 1.47–5.20, *P* = 0.0005) (Fig. 4A). Additionally, the funnel plot appeared approximately symmetrical (Fig. 4B), suggesting no obvious publication bias. It is noteworthy that some studies (Sharma *et al*. 2021; Sun *et al*. 2018; Wang *et al*. 2023) have investigated the impact of exosomes on islet proliferation and cell death markers. The findings indicate that exosomes can facilitate islet proliferation and attenuate islet cell death (Table 2).

**Table 2:** The effect of SC-Exs on proliferation and cell death markers in diabetic islets

Study	Proliferation indicators				Cell death indicator		
	Brdu	PCNA	Ki-67		Caspase 3	Caspase 9	Ferroptosis
Sharma *et al*. 2021	↑						
Sun *et al*. 2018		↑			↓		
Wang *et al*. 2023			↑			↓	↓
↑, indicator levels was increased after SC-EX treatment; ↓, indicator levels was reduced after SC-EX treatment							

### Stem cell-derived exosomes may reduce inflammatory response in experimental animals

Five studies have reported the effects of SC-Ex on inflammatory markers in experimental animals (Kashani *et al*. 2023; Kouhestani *et al*. 2023; Nojehdehi *et al*. 2018; Ou *et al*. 2024; Wang *et al*. 2023), including anti-inflammatory cytokines (IL-10, IL-4 and TGF-β1) and pro-inflammatory cytokines (IL-6, IL-17, IFN-γ, IL-1β, and NF-κB). The results demonstrated that the levels of anti-inflammatory cytokines in experimental animals were increased following SC-Ex treatment, whereas the levels of pro-inflammatory cytokines were decreased (Table 3). Because most indicators were only involved in one or two studies, we conducted a meta-analysis only for the anti-inflammatory cytokine IL-10. Given the considerable heterogeneity, a random-effects model was employed for the meta-analysis. The results showed that exosomes exhibited a tendency to elevate the level of IL-10 in experimental animals (supplementary Fig. S2).

**Table 3:** The effect of SC-Exs on inflammatory cytokines in diabetic islets

Study	Anti-inflammatory cytokines				Pro-inflammatory cytokines					
	IL-10	IL-4	TGF-β1		IL-6	IL-17	IL-1	IFN-γ	IL-1β	NF-κB
Kashani *et al*. 2023	↑	↑	→		↓				↓	
Kouhestani *et al*. 2023	↑								↓	↓
Nojehdehi *et al*. 2018	↑	↑	↑			↓		↓		
Ou *et al*. 2024	↑	↑					↓	↓		
Wang *et al*. 2023								↓		
↑, cytokine levels was increased after SC-EX treatment; ↓, cytokine levels was reduced after SC-EX treatment; →, no significant change										

## DISCUSSION

Diabetes is a metabolic disease characterized by chronic hyperglycemia, for which no curative therapy is currently available. As the disease progresses, pancreatic islets undergo profound structural and functional alterations. Diabetic islets frequently exhibit elevated inflammatory mediators (Böni-Schnetzler and Meier 2019), increased fibrosis (Tsai *et al*. 2021), extensive amyloid deposition (Westermark *et al*. 2011), and diminished vascularization (Okajima *et al*. 2022). These pathological changes disrupt the islet microenvironment, ultimately leading to β-cell loss and a marked reduction in insulin secretion. Restoring islet function has become a key objective in the treatment of diabetes, as it holds the potential to address the underlying dysfunctions driving the disease.

Exosomes are nanosized extracellular vesicles (40–160 nm in diameter) secreted by various cell types, including tumor, immune, and neuronal cells. They mediate intercellular communication by transferring proteins, RNAs, and lipids, thereby regulating a wide range of biological processes (Kalluri and LeBleu 2020). In the tumor microenvironment, exosomes promote cancer growth, metastasis, and immune evasion (Hu *et al*. 2020; Paskeh *et al*. 2022). Moreover, they are involved in immune regulation by transporting inflammation-related molecules that modulate immune activation and suppression, contributing to chronic inflammation and autoimmune diseases such as diabetes and inflammatory bowel disease (Anel *et al*. 2019; Lin *et al*. 2024b; Zhang *et al*. 2019; Zheng *et al*. 2024).

Due to their natural biocompatibility, low immunogenicity, and ability to cross cell membranes, exosomes have emerged as promising carriers for drug delivery and gene therapy. They can efficiently transport small molecules, nucleic acids, and proteins to target cells with minimal toxicity (Liang *et al*. 2021). For instance, exosomes derived from brain endothelial cells successfully delivered doxorubicin across the blood–brain barrier and inhibited tumor growth in zebrafish models (Yang *et al*. 2015). Similarly, exosomes have been used to deliver other therapeutic cargos, including small RNAs and CRISPR/Cas9 components, demonstrating their potential in diverse disease contexts (Didiot *et al*. 2016; Fuhrmann *et al*. 2015; Kobayashi *et al*. 2020; Lee *et al*. 2016; Ye *et al*. 2020).

With respect to islet biology, several studies have shown that exosomal components can exert both protective and detrimental effects on β-cells. Milk-derived exosomal miRNAs were found to promote β-cell dedifferentiation, impair insulin secretion, and suppress autophagy (Melnik 2019). In diabetic models, β-cell-derived exosomal miR-21, miR-26a, miR-320b, and miR-23a-3p induced β-cell apoptosis and reduced serum insulin levels (Song *et al*. 2023; Wang *et al*. 2024; Xu *et al*. 2020). Circulating exosomes from obese individuals inhibit β-cell proliferation through inflammatory signaling (Xie *et al*. 2019), while lymphocyte-derived exosomes may trigger autoimmune responses contributing to type 1 diabetes (Guay *et al*. 2019).

**Figure 5:**
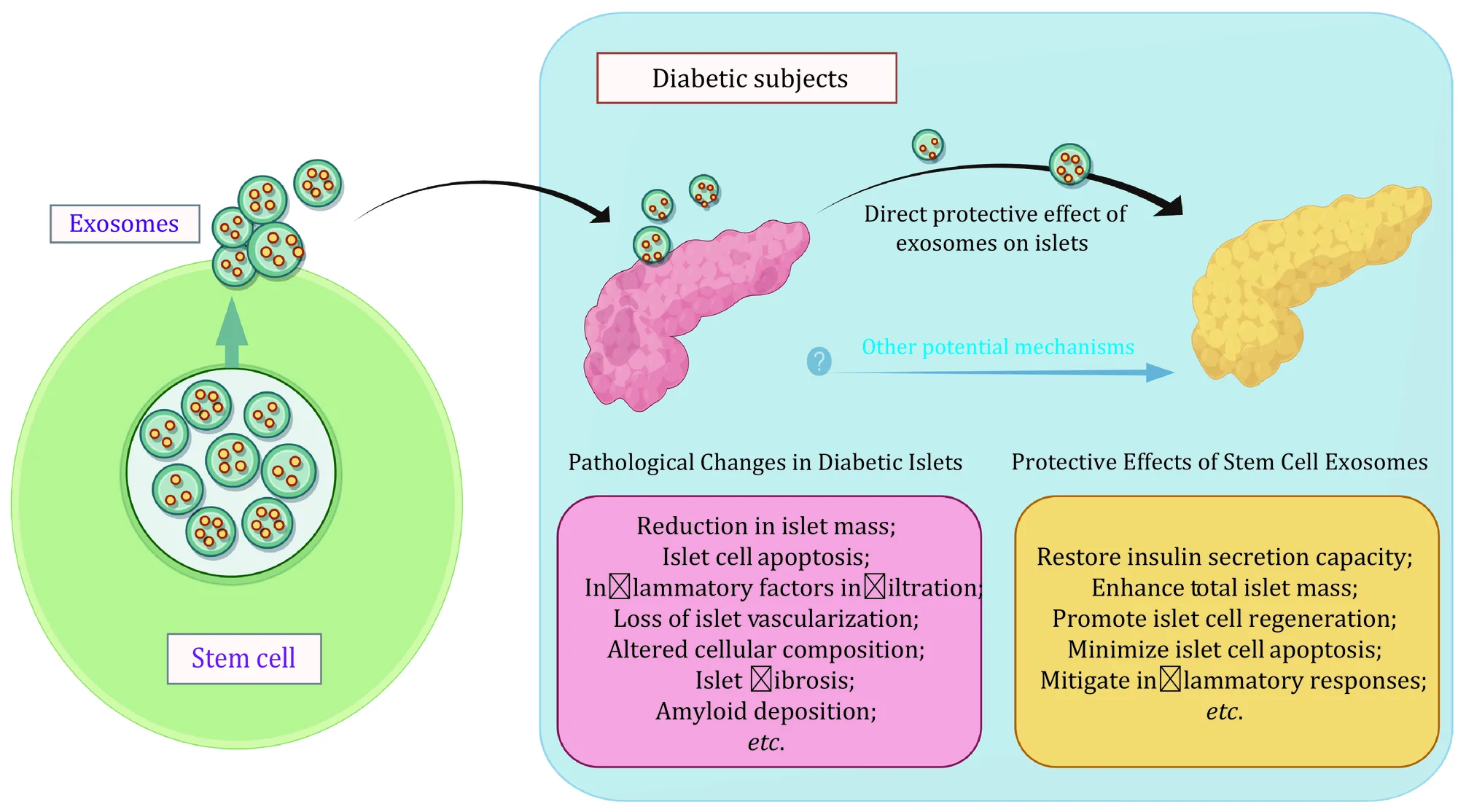
Diagram of islet damage mechanisms and the protective role of SC-Exs on islets in diabetes. The left section illustrates the pathological changes in diabetic islets, including reduced islet mass, islet cell apoptosis, inflammation, loss of islet vascularization, altered cellular composition, fibrosis, and amyloid deposition. The right section highlights the protective effects of stem cell exosomes, including restoring insulin secretion capacity, enhancing total islet mass, promoting islet cell regeneration, minimizing islet cell apoptosis, and mitigating inflammatory responses. This figure was created with Figdraw

In this context, we systematically evaluated the effects of stem cell-derived exosomes (SC-Exs) on diabetic islet function to explore their therapeutic potential. Although no clinical trials were identified, ten eligible preclinical studies were included. The pooled analysis revealed that SC-Exs significantly increased insulin levels and islet numbers in diabetic animals compared with controls. Moreover, SC-Exs promoted islet proliferation, inhibited cell death, and alleviated inflammatory responses (Fig. 5, created with Figdraw). To our knowledge, this is the first meta-analysis focusing on the impact of SC-Exs on islet function.

SC-Exs may preserve diabetic islet structure and function through both direct pancreatic actions and indirect extra-pancreatic mechanisms. Directly, SC-derived exosomes have been summarized to support β-cell survival and regenerative responses (*e*.*g*., promoting proliferation and limiting apoptosis) and to modulate inflammatory cues relevant to islet integrity, consistent with the direction of our pooled islet outcomes. Indirectly, *in vivo* tracking studies indicate that systemically administered exosomes predominantly accumulate in clearance/metabolic organs such as the liver and spleen (Wiklander *et al*. 2015), implying that part of the benefit may arise from actions outside the pancreas. Consistent with this, adipose-derived stem cell (ADSC) exosomes improved metabolic homeostasis in diet-induced obese mice, including enhanced insulin sensitivity and reduced hepatic steatosis, while driving M2 macrophage polarization and “beiging” in white adipose tissue — changes that would be expected to lower systemic insulin resistance and thus reduce β-cell workload (Zhao *et al*. 2018). Similarly, Wharton’s jelly MSC (WJMSC) exosomes have been shown in obese models to reduce macrophage infiltration and pro-inflammatory cytokine expression in both liver and adipose tissue with accompanying improvements in insulin resistance, again supporting an indirect route to islet protection through systemic anti-inflammatory and insulin-sensitizing effects (Kim *et al*. 2024). At the cellular level, human umbilical cord-derived mesenchymal stem cell (hUC-MSC) exosomes can also enhance insulin-stimulated glucose uptake and insulin-signaling activity in insulin-resistant adipocytes, further supporting adipose tissue as a plausible extra-pancreatic target contributing to secondary islet benefit (Chen *et al*. 2021).

This meta-analysis has several limitations that should be considered when interpreting the findings. First, the number of included studies was limited, and several studies had small sample sizes, which may affect the precision and generalizability of the pooled estimates. Second, reporting was incomplete in some studies, with insufficient methodological or outcome details, which restricted data extraction and may have introduced uncertainty. Third, substantial heterogeneity was observed across outcomes, likely due to differences in animal species, diabetes models, exosome sources, dosing regimens, follow-up durations, and outcome measurements. Formal subgroup analyses were not feasible for most outcomes because of the limited number of studies per subgroup. Heterogeneity was quantified using Cochran’s *Q*-test and the *I*^2^-statistic, and random-effects models were applied when heterogeneity was substantial. To further explore and manage heterogeneity, we used Galbraith plots to identify potential outliers and conducted leave-one-out sensitivity analyses to evaluate the robustness of the pooled estimates. Where appropriate, outlier studies were excluded in exploratory analyses, and the direction of effect remained consistent while heterogeneity was reduced. Collectively, these steps support the stability of our conclusions despite between-study variability.

Despite these limitations, this study has notable strengths, including (1) a comprehensive search across four databases, (2) clearly defined inclusion and exclusion criteria, (3) standardized data extraction procedures, (4) rigorous risk-of-bias assessment, and (5) being, to our knowledge, the first meta-analysis focusing on stem cell-derived exosomes and their effects on islet function.

## CONCLUSION

In this study, we conducted a meta-analysis to evaluate the effects of SC-Exs on islet function in the context of diabetes. The results demonstrated that SC-Exs exert significant protective effects on islet function. By promoting islet cell proliferation, inhibiting cell death, and reducing the expression of inflammatory factors, these exosomes markedly improved diabetes-related islet dysfunction. These findings provide novel insights and potential therapeutic strategies for diabetes treatment, suggesting that SC-Exs could serve as a promising therapeutic tool with clinical potential. However, further research is needed to elucidate the underlying mechanisms and to assess their safety and efficacy in clinical settings.

## METHODS

This meta-analysis was performed in accordance with the Preferred Reporting Items for Systematic Reviews and Meta-Analysis (PRISMA) reporting guidelines (Page *et al*. 2021). The current meta-analysis was registered on the International Prospective Register of Systematic Reviews (PROSPERO) under the registration number CRD420251055493.

### Search strategy

A systematic search of PubMed, Cochrane Library, Web of Science, and Embase was conducted for relevant studies evaluating the efficacy of exosome application in diabetic animals, published from inception to May 1, 2025. The search terms employed were “exosome*”, “vesicle*”, “microparticle*”, “nanovesicle*”, “islet*”, “β*cell*”, “beta cell*”, “diabetes mellitus”, “diabet*”, “T1DM”, and “T2DM”. Furthermore, the references from these relevant articles were carefully examined for additional eligible studies.

### Eligibility criteria

Studies were included in this meta-analysis when they adhered to the following criteria: (1) The study was conducted *in vivo*, utilizing diabetic animal models or human patients; (2) The study described a detailed procedure for the identification and extraction of exosomes; (3) The treatment group used only stem cell-derived exosomes, while the control group used PBS or no treatment at all; (4) One or more outcomes were reported in the publications, including insulin secretion levels, islet cell death or proliferation indicators, and inflammatory markers. Studies were excluded from this meta-analysis when they adhered to the following criteria: (1) The studies were conducted *in vitro*; (2) The articles were not published in the English language; (3) The treatment group utilized exosomes that were not from stem cells or combined with other materials; (4) The control group used other dressings; (5) The studies were retracted or presented duplicate data; (6) The studies were correspondence letters, case reports, reviews, *etc*.; (7) The data were not provided or extractable.

### Data extraction

Two investigators (Y. Jiang, L. Chen) extracted the following data independently from the entire manuscripts using a standard form: lead author, publication year, characteristics of the animal models, source of exosomes, dosage, duration of follow-up, and pertinent outcomes of the islet function. Any discrepancies between the two investigators were resolved through discussion with a third author (T. Z.).

### Quality assessment

The Systematic Review Centre for Laboratory Animal Experimentation’s (SYRCLE’s) risk of bias tool was employed by two independent authors (Z. D. and X. L.) to evaluate the quality of each included study. The third author (Y. H.) will be adjudicated in case of any dispute. The quality of included study trials in this tool was assessed using a comprehensive range of methodological criteria, including sequence generation (selection bias), baseline characteristics (selection bias), allocation concealment (selection bias), random housing (performance bias), blinding (performance bias), random outcome assessment (detection bias), blinding (detection bias), incomplete outcome data (attrition bias), selective outcome reporting (reporting bias), and other sources of biases.

### Statistical analysis

The data were analyzed using Stata 14.0 and Review Manager (RevMan) 5 software. For continuous variables, the mean and standard deviation (SD) were extracted, and the results were expressed as standardized mean differences (SMDs) with 95% confidence intervals (CIs). Statistical heterogeneity was quantified using Cochran’s *Q*-test (*P* < 0.10 indicating significant heterogeneity) and the *I*^2^-statistic (with *I*^2^-values of ~25%, ~50%, and ~75% reflecting low, moderate, and high heterogeneity, respectively). A fixed-effects model was used when heterogeneity was low; otherwise, a random-effects model was applied to account for between-study variability. To identify and manage potential sources of heterogeneity, we generated Galbraith (radial) plots to detect outlying studies contributing disproportionately to heterogeneity, we then conducted leave-one-out sensitivity analyses by sequentially omitting each study to evaluate the robustness of pooled estimates and the influence of individual studies on *I*^2^ and effect sizes. When outliers were detected, exploratory analyses excluding these studies were performed to assess whether conclusions were driven by single studies; results were compared with the primary analysis to ensure consistency in the direction and magnitude of effect. Publication bias was assessed by visual inspection of funnel plots.
